# Building Valveless Impedance Pumps From Biological Components: Progress and Challenges

**DOI:** 10.3389/fphys.2021.770906

**Published:** 2022-01-31

**Authors:** Narine Sarvazyan

**Affiliations:** Department of Pharmacology and Physiology, School of Medicine and Health Science, The George Washington University, Washington, DC, United States

**Keywords:** valveless pumping, Liebau mechanism, tissue engineering, biofabrication, heart development

## Abstract

Valveless pumping based on Liebau mechanism entails asymmetrical positioning of the compression site relative to the attachment sites of the pump’s elastic segment to the rest of the circuit. Liebau pumping is believed to play a key role during heart development and be involved in several other physiological processes. Until now studies of Liebau pump have been limited to numerical analyses, *in silico* modeling, experiments using non-biological elements, and a few indirect *in vivo* measurements. This review aims to stimulate experimental efforts to build Liebau pumps using biologically compatible materials in order to encourage further exploration of the fundamental mechanisms behind valveless pumping and its role in organ physiology. The covered topics include the biological occurrence of Liebau pumps, the main differences between them and the peristaltic flow, and the potential uses and body sites that can benefit from implantable valveless pumps based on Liebau principle. We then provide an overview of currently available tools to build such pumps and touch upon limitations imposed by the use of biological components. We also talk about the many variables that can impact Liebau pump performance, including the concept of resonant frequencies, the shape of the flowrate-frequency relationship, the flow velocity profiles, and the Womersley numbers. Lastly, the choices of materials to build valveless impedance pumps and possible modifications to increase their flow output are briefly discussed.

## Brief History

**FIGURE 1:**
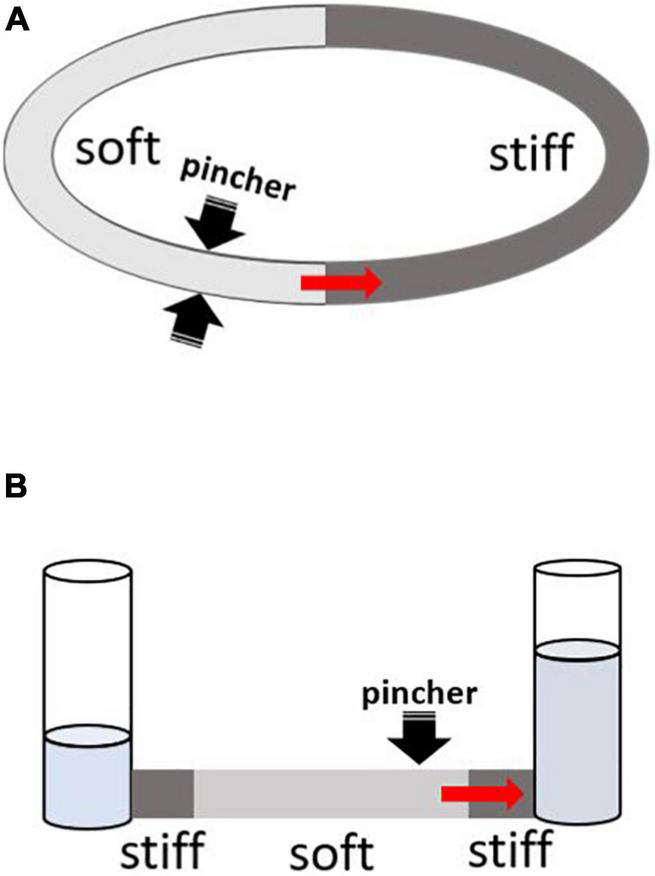
Two main configurations for testing Liebau-based pumping. The red arrow depicts the direction of mean flow as originally reported by G. Liebau from pincher toward the nearest junction with stiff tubing. Depending on the compression frequency, the flow can also occur in an opposite direction. **(A)** Two tubes with different compliances are connected to form a close loop conduit. Pincher is typically a rectangular piston being pushed into soft tubing (light gray) by some form of linear actuator. When the asymmetrically positioned pincher is periodically compressed, it creates pulsative flow. **(B)** The compliant segment is depicted in light gray. On both ends, it connects to stiff tubes that are attached to open tanks filled with fluid. Pincher is an asymmetrically positioned piston that compresses the soft segment with a defined frequency.

About 70 years ago, German physician Gerhard Liebau came up with a new concept of valveless pumping (Liebau, 1954, 1955). It involves a periodic compression of a compliant tube connected to a stiffer tubing on both ends (Figure 1). Asymmetric positioning of the pincher is required to generate the flow, and the relationship between the pinching frequency and the flow is highly non-linear. This mechanism acquired the name of a “Liebau-pump” or a “Liebau-based” principle. Due to a common assumption that it is a mismatch in impedance at the junctions between compliant and stiff segments that generates net flow, the Liebau pump has also been referred to as a “valveless impedance pump.”

Initially, the physiological significance of the Liebau pump was largely hypothetical; therefore, for the next 50 years, this concept remained of interest mainly to physicists (Thomann, 1978; Jung and Peskin, 2002; Borzì and Propst, 2003; Ottesen, 2003; Auerbach et al., 2004; Jung, 2007). However, in 2006, a landmark paper by Gharib’s group provided the first direct experimental evidence that the Liebau-type mechanism might be involved in driving blood flow *in vivo* (Forouhar et al., 2006). The authors’ conclusions were based on tracking erythrocyte movement in the hearts of live zebrafish embryos. The data suggested that the observed flow pattern was more consistent with the Liebau pumping mechanism than that based on peristalsis. Modification of the Liebau pump that included an additional gelatinous layer was soon proposed to explain the physiological role of cardiac jelly in helping drive blood flow in embryonic hearts (Loumes et al., 2008). Additional modifications that mimic possible biological scenarios and further increase flow efficiency, have also been suggested. These included inclusion of bends (Hiermeier and Männer, 2017), insertion of cavities (Kozlovsky et al., 2015), use of tapered connectors (Lee et al., 2017), or asymmetric arrangement of resistances between the two sides of the compliant tube (Wen and Chang, 2009). Due to its complexity and dependence on many variables, the pumping mechanism proposed by Liebau remains the subject of interest across a wide range of disciplines, including physiology, engineering, physics, and biomedical research (Hickerson et al., 2005; Rinderknecht et al., 2005; Hickerson and Gharib, 2006; Manopoulos et al., 2006, 2020; Bringley et al., 2008; Timmermann and Ottesen, 2009; Rosenfeld and Avrahami, 2010; Meier, 2011; Lee et al., 2013; Kozlovsky et al., 2016; Zislin and Rosenfeld, 2018; Li et al., 2019; Davtyan and Sarvazyan, 2021).

## Biological Occurrence of Liebau Pumps

Today, the possibility of the physiological existence of Liebau-like pumps is rarely disputed, with ongoing discussions as to what degree embryonic heart tubes function as peristaltic vs. impedance-based pump (Männer et al., 2010). A number of papers have suggested that it is the combination of the two mechanisms that is involved (Santhanakrishnan et al., 2009; Santhanakrishnan and Miller, 2011; Kozlovsky et al., 2016). Indeed, at the end of the first month, the heart of the human embryo is still valveless, yet its beating yields unidirectional blood flow with a non-linear flow-frequency pattern (Forouhar et al., 2006). Behavior of an embryonic heart can be seen as a stage of early evolutionary development since the valveless circulatory system has been described for several classes of invertebrates and some lower vertebrates (Anderson, 1981). It was also suggested that circulation of cerebrospinal fluid (Longatti, 2018) as well as blood flow caused by compressions during cardiopulmonary resuscitation (Ottesen, 2003) can be explained, at least in part, by Liebau-type pumping. There is also an indirect evidence that the latter contributes to blood flow in other body locations, such as an aorta (Pahlevan and Gharib, 2013).

## Main Differences Between Peristaltic and Liebau Pumps

There are number of fundamental differences that distinguish the two types of valveless pumps found in Nature. Peristaltic pumps belong to the class of so-called positive displacement pumps. Contraction waves passing through the wall of the vessel lead to lumen compression, which squeezes the content of the vessel in the same direction as the contraction wave. As a result, continuous flow is generated with peak flow velocity equal to the speed of the propagating contraction wave. Peristaltic pumps exhibit a linear relationship between the compression frequency and the flowrate (Jaffrin and Shapiro, 1971).

The behavior of a Liebau pump exhibits a number of key differences. Only a small segment of the wall is actively compressed, with the rest of the pressure wave passively propagating through the vessel. Instant flow pattern is pulsatile with direction of flow changing back and forth within each compression event. The mean flowrate can be significant even in the absence of full lumen closure. When recorded over a wide range of compression frequencies, the relationship between the frequency and the mean flow rate is non-linear, including reversals in the direction of flow (Liebau, 1955).

## Potential Uses of Liebau Pumps Made From Biological Components

As of today (with two exceptions detailed below), studies of Liebau pumps have been limited to numerical analyses, *in silico* modeling, experiments using non-biological elements, and a few indirect *in vivo* measurements. Therefore, there is a need for experimental studies that recreate Liebau pumps from biological materials using tissue engineering methods, biofabrication tools or biomimetric approaches. Such efforts can yield multiple benefits. First, they will lead to a better understanding of physiological mechanisms responsible for blood flow generation during heart development, including cases of collapsed flow caused by cardiac malformations or genetic defects. Second, the creation of biofabricated Liebau pumps (abbreviated as BLP thereafter) will be of interest from a purely biological perspective. There is ample evidence that Liebau-type pumping is involved in the circulation of fluids in a wide variety of biological forms, including both vertebrates and invertebrates (Johansen et al., 1980) and it will be quite interesting to look into the evolutionary adaptation of these valveless pumps. Third, miniature self-beating BLPs can be used to circulate fluids in organ-on-a-chip or human-on-chip platforms (Zhang et al., 2018; Ma et al., 2021). Lastly, in the long term, one can envision the clinical use of biomimetic devices, such as those based on the Liebau principle, that will help drive the flow of fluids at various locations within the human body (Dai et al., 2006; Sarvazyan, 2014a,b; Swift et al., 2014). The next paragraph will consider the later possibilities in slightly more detail.

## Possible Body Sites That Can Benefit From Implantable Biofabricated Liebau Pumps

There are multiple types of fluids in the human body, including blood, lymph, saliva, gastric juice, semen, urine, tears, and many others. Some fluids are delivered by simple secretion into the target compartment or a duct, while others are delivered by active circulation. The most obvious instance of the latter is blood that circulates due to active pumping by the beating heart aided by contraction of skeletal muscle that surrounds valve-containing veins. Another example is a lymph flow, which is enabled by the presence of one-way valves and repetitive contractions of smooth muscle within walls of lymphatic vessels (Zawieja, 2009), again aided by compression by the surrounding skeletal muscle. An additional case is semen ejection, which is caused by contraction of smooth muscle within walls of vas deferens. One can also mention peristalsis of smooth muscle in the walls of the ureters that moves urine toward the urinary bladder. When, for a variety of reasons, these physiological pumping mechanisms become impaired, one can envision the use of BLP-based therapies to either aid or restore the flow. Importantly, the key components of BLPs can be added without disrupting the integrity of the vessel, and there are no requirements to create one-way valves. These key components can be positioned outside the vessel of interest and include a pair of low compliance cuffs and a band of periodically contracting muscles. The latter can be made from a patient’s induced pluripotent stem cell (iPS) derived cardiomyocytes, or a stimulable ring of skeletal muscle cells. The most attractive aspect of such a design is that the integrity of the inner endothelial layer does not have to be disrupted, avoiding possible fibrosis, blockage, or, in case of blood flow, thrombi formation.

## Available Biofabrication Tools

The last two decades have led to an explosion of biofabrication tools and approaches. It is now possible to build cell-free or cell-seeded vessels with different degrees of elasticity and compliance (Schuurman et al., 2011; Nguyen et al., 2016). Tissue engineered cardiac and skeletal muscle strips have been developed (Juhas et al., 2014). Initially, force generation by these engineered muscle constructs was quite low (Zimmermann et al., 2002), yet the use of electrical and mechanical stimulation (Radisic et al., 2004; Tandon et al., 2009; Khodabukus et al., 2019), enhanced perfusion (Carrier et al., 1999), and seeding cells into stretchable scaffolds (Zhang et al., 2013) or around suture templates (Nunes et al., 2013) led to a significant increase in the force of contraction that such engineered muscles can create. Tissue engineered muscle strips can now be developed from patient-specific iPSCs (Karabekian et al., 2015a; Breckwoldt et al., 2017) alleviating concerns of immunorejection when such constructs are implanted back into the patient (Karabekian et al., 2015a,b). Another set of useful tools comes from the optogenetic field. One can now repetitively stimulate muscle constructs made from cells that express light-sensitive channels using pulses of light (Burton et al., 2015; Raman et al., 2016; Entcheva and Kay, 2020). Optogenetics also offers the possibility of a programmable spatiotemporal coordination of excitability along the tissue engineered muscular construct (Entcheva, 2013). Ability to 3D-bioprint multicellular multilayered constructs (Cvetkovic et al., 2014; Kang et al., 2016; Zhang et al., 2017; Koti et al., 2019) is yet another tool that can be used to create BLPs. A particularly promising approach for this task is additive-lathe 3D printing, an approach that uses rotating cylindrical mandrel to bioprint tubular constructs (Reeser and Doiron, 2019).

## Published Attempts to Create Functional Biofabricated Liebau Pumps

The design shown in Figure 1A is deceivingly simple. In fact, creating any sizable pressure or flow using Liebau pump built from biological elements has been a real challenge. To the best of our knowledge, as of today there is just one published peer-reviewed attempt to create a BLP (Li et al., 2019). Yet even this study created only a partial BLP, with its compression element made from biological material while the vessel parts were not. Specifically, Li et al. (2019) examined the ability of a ring formed by differentiated cells from mouse myoblast cell line C2C12 to create flow in a polyacrylamide-based compliant tube inserted into a more rigid PDMS mold. Repetitive compression of the tube by the tissue engineered muscle led to a 0.3–1% decrease in tube radius, yielding mean flowrates of < 0.4 μL/s. Non-peer reviewed attempts to build BLPs can also be found in the PhD thesis of Hesham Azizgolshani from Gharib’s lab (Azizgolshani, 2013). These studies attempted to create BLPs using neonatal rat cardiac myocytes seeded onto vessels made from decellularized small intestinal submucosa. Cell contraction led to < 2% change in tube radius yielding < 20 nL/s mean flowrate.

The very low mean flow rates seen in these two studies (Azizgolshani, 2013; Li et al., 2019) are troublesome for two reasons. First, they offer little use for any real flow improvement in physiologically relevant settings. Second, they are in sharp contrast with much higher flow rate values obtained when using non-biological components of Liebau pumps. In the next section, we will consider factors that may limit the efficiency of BLPs, followed by the sections that consider ways to circumvent or minimize these limitations.

## Limitations Imposed by the Use of Biological Components

There are several reasons behind the low flow rates reported by the above-mentioned attempts to build BLPs. The same reasons are likely to play a role in any future attempts to create Liebau pumps using biological components, particularly from mammalian cells and more specifically from their human counterparts.

The first reason is that, when compared to mechanical actuators, the pincher segment of a BLP can operate within a very limited range of compression frequencies. Cardiac myocytes isolated or derived from iPSC of animals with intrinsically high heart rates, such as mice, can be continually paced at frequencies up to 7–8 Hz (Bers, 2001). Their human counterparts are unlikely to go over 3 Hz. In most cases of skeletal muscle-based constructs, stimulation frequency can be increased to 10 Hz (Khodabukus et al., 2019), after which contractions become fused, leading to tetanus. Maximal frequency of smooth muscle contractions varies depending on muscle subtype and location, but in general, due to different molecular mechanisms underlying excitation-contraction coupling and relaxation cycles of smooth muscle, these cells are too slow to be viable candidates for repetitive fast contractions. To conclude, compression frequencies by a BLP pincher can hardly exceed 10 Hz, with 1–4 Hz being the most realistic range.

This leads us to the second reason: the limited degree of muscle shortening. By excluding smooth muscle as a main component of a BLP pincher, one is left with cross-striated muscle candidates, such as cardiac and skeletal muscle. Cross-striated muscle can shorten to only 15–20% of its resting length (Bers, 2001). Therefore, a pincher made from a muscular ring of cross-striated muscle has a limited ability to decrease the lumen. This limitation can be overcome by a non-conventional way of arranging muscle elements—some of which will be considered later. Notably, in previously mentioned studies by Azizgolshani (2013) and Li et al. (2019), decreases in lumen diameter were much smaller than the above-mentioned limit of 15–20%, leading us to the third reason.

The third reason involves the way in which the muscle layer is created. Both above-cited studies used a simple cell seeding approach to create a ring of tissue-engineered muscle (Zimmermann et al., 2002). It is now well recognized that such an approach yields a weakly contracting muscle due to underdeveloped connections between the cells and the lack of a well-organized sarcomere structure inside the cells. The use of preconditioned tissue-engineered muscle strips, strength of which has been increased by electrical and mechanical stimuli (Tandon et al., 2009; Nunes et al., 2013; Ronaldson-Bouchard et al., 2019) should significantly improve the outcome of BLPs. An additional consideration should be given to a spiral arrangement of multiple fibers within the pincher. The latter has been shown to greatly increase the efficiency of cardiac ejection (Torrent-Guasp et al., 2001) and is likely to do the same for the efficiency of the BLP pincher.

The fourth and the last reason why reported BLP yielded such low flow rates is that their design did not take into consideration insights from theoretical and experimental studies that used non-biological components. In the next sections, we will review available experimental reports and a few numerical studies with the goal of outlining strategies for more effective implementation of Liebau pumps using biofabrication tools. We will start by listing different parameters that can influence the performance of the Liebau pump.

## Variables That Impact Liebau Pump Performance

Despite its apparent simplicity, the physics behind flow and pressure generation by a Liebau pump is quite complex and, as such, remains the subject of intense interest by several generations of physicists and mathematicians (Takagi and Takahashi, 1985; Borzì and Propst, 2003; Manopoulos et al., 2006; Jung, 2007; Loumes et al., 2008; Kozlovsky et al., 2015). Because of its complexity, all modeling studies had to omit many involved variables. The list of such variables, each of which can potentially affect the mean flow rate created by a Liebau pump, can be seen in Table 1. They can be divided into four categories. The first category describes the overall pump design, including the tubing being closed or open, number of pinchers, presence of kinks or gelatinous inner layer, or difference in resistances by the two stiff ends. The second category includes the physical properties of compliant tube segments (notably, the impact of the physical properties of stiff tube segments remains to be explored). The third category includes properties of the pincher, such as its length, degree of vessel occlusion, its relative position with respect to the two ends of the compliant segment, compression frequency, duty cycle, and its dynamic waveform (i.e., square form vs. sinusoid). The fourth category includes properties of the fluid, including density, viscosity, and fluid volume, the latter having a direct impact on transmural pressure and therefore compliance of the elastic segment. For readers wanted to learn more, Table 1 lists studies that considered, either numerically or experimentally, the impact of specific variables. Notably, the non-linear character of Liebau pumps precludes predicting, in a simple manner, the effect of many variables. Therefore, changing the frequency of pinching from, for example, 10–20 Hz for the Liebau pump with one set of dimensions will increase the flow, while it will decrease it for the pump with different geometry. Such non-linearity and involvement of multiple variables explains a vast range of measured flow rate values created by experimental variations of the Liebau pump (Table 2).

**TABLE 1:** Major variables that affect performance of Liebau pump.

Variable	*In vitro*	*In silico*	Combined
**Group 1: Design**			
Open vs. close	Rinderknecht et al., 2005		
Double wall	Hiermeier and Männer, 2017	Loumes et al., 2008	
Kinks	Hiermeier and Männer, 2017		
Tapering	Lee et al., 2017		
Branching		Rosenfeld and Avrahami, 2010; Zislin and Rosenfeld, 2018	
Buckle	Li et al., 2019		
Added cavities		Kozlovsky et al., 2015	
**Group 2: Tube**			
Length	Rinderknecht et al., 2005	Manopoulos et al., 2006; Zislin and Rosenfeld, 2018	Timmermann and Ottesen, 2009
Diameter	Hickerson et al., 2005; Rinderknecht et al., 2005	Zislin and Rosenfeld, 2018	
Wall thickness	Bringley et al., 2008	Jung, 2007	
Elastic modulus		Jung, 2007	
Loop resistance	Hickerson et al., 2005		
Shape	Wen and Chang, 2009		
**Group 3: Pincher**			
Width	Hickerson et al., 2005; Manopoulos et al., 2020	Manopoulos et al., 2006; Rosenfeld and Avrahami, 2010; Zislin and Rosenfeld, 2018	
Position	Hickerson et al., 2005; Bringley et al., 2008; Wen and Chang, 2009; Lee et al., 2013; Hiermeier and Männer, 2017; Manopoulos et al., 2020; Davtyan and Sarvazyan, 2021	Borzì and Propst, 2003; Rosenfeld and Avrahami, 2010; Zislin and Rosenfeld, 2018	Ottesen, 2003; Hickerson and Gharib, 2006; Timmermann and Ottesen, 2009
Force profile		Jung, 2007; Zislin and Rosenfeld, 2018	
% Occlusion	Hickerson et al., 2005; Wen and Chang, 2009; Manopoulos et al., 2020	Manopoulos et al., 2006; Jung, 2007; Rosenfeld and Avrahami, 2010; Kozlovsky et al., 2016	Ottesen, 2003
Duty cycle	Hickerson et al., 2005		
# Of pinchers	Lee et al., 2013		
**Group 4: Fluid**			
Fluid viscosity	Meier, 2011; Hiermeier and Männer, 2017; Davtyan and Sarvazyan, 2021		Timmermann and Ottesen, 2009
Fluid density	Hiermeier and Männer, 2017; Davtyan and Sarvazyan, 2021		Timmermann and Ottesen, 2009
Fluid volume	Hickerson et al., 2005		

**TABLE 2:** Flow rate values created by experimental variations of the Liebau pump.

Year	First author	References #	Setup	Variables tested	Provided or estimated from the cited study								Calculated based on Eq. 1 and 3				
					*L*	*ID*	*h*	*E*	*F range*	∼*Fmax*	*w*	*Qmax*	*h/D*	*c*	*c’*	*Fn*	*Qprst*

Non-biological					*cm*	*cm*	*cm*	*MPa*	*Hz*	*Hz*	*cm*	*ml/s*		*m/s*	*m/s*	*Hz*	*ml/s*
2003	Otteson	Ottesen, 2003	Closed	Frequency, pincher location, compression ratio	50	2.00	0.100	0.41	2.5–3.5	2.9	1.00	1.20	0.050	4.5	5.2	5	9.1
2005	Hickerson	Hickerson et al., 2005	Open	Frequency, elasticity, pincher size and location, system size, transmural pressure	15	1.91	0.079	1.19	1–8	4.7	2.54	40	0.042	7.0	8.1	23	34
			Closed		2	0.19	0.005	0.22	20–142	55	0.24	0.15	0.026	2.4	2.8	60	0.37
2005	Rinderknecht	Rinderknecht et al., 2005	Closed	Frequency, open vs. closed, open loop dimensions	1.96	0.20	0.005	0.40	1–180	55	0.24	0.18	0.025	3.2	3.6	81	0.41
			Open		1.20	0.025	0.005	2.00	82	82	0.04	0.0003	0.200	20.0	23	833	0.0016
2006	Hickerson	Hickerson and Gharib, 2006	Closed	Frequency, transmural pressure	15	2.00	0.080	1.19	0–8	5	2.50	20	0.040	6.9	7.9	23	37
2008	Bringley	Bringley et al., 2008	Closed	Frequency, pincher location, elastic tube rigidity	17	1.90	0.05	0.99	0.5–6.8	6.8	2.22	60	0.027	5.2	5.9	15	43
2009	Wen	Wen and Chang, 2009	Open	Pincher location, symmetry, degree of compression, cross section shape	5	0.60	0.100	2.20	8–40	26	3.00	10	0.167	19.1	22	191	22
2011	Meier	Meier, 2011	Closed	Resistance, transmural pressure, wall thickness, pincher location, amplitude and offset of excitation	2	0.18	0.005	1.00	1–140	60	0.28	0.05	0.028	5.3	6.1	132	0.43
					2	0.15	0.025	13.50	1–140	140	0.28	0.14	0.167	47.4	54.5	1186	0.69
2013	Lee	Lee et al., 2013	Open	Frequency, pincher location	50	2.80	0.200	2.16	4–7	4.5	5.00	127	0.071	12.4	14.3	12	138
2017	Hiermeier	Hiermeier and Männer, 2017	Open	Frequency, viscosity, straight vs. kink, double walled	34.8	0.63	0.013		0.5–3	3	1.1	0.5	0.021				1.03
2020	Manoupolus	Manopoulos et al., 2020)	Closed	Frequency, cross-sectional area, pincher size & location	100	1.20	0.100	1.96	1–12	9.3	10.00	83	0.083	12.8	14.7	6	105
2021	Davtyan	(Davtyan and Sarvazyan, 2021	Closed	Frequency, pincher location, viscosity	3.8	0.50	0.10	0.05	0.5–2.5	2.5	0.35	0.04	0.200	3.1	3.5	40	0.17
**Biological**																	
2006	Forouhar	Forouhar et al., 2006	Closed	Frequency	0.02	0.006	0.002	0.002	1.6–3	2	0.00	1.0E–06	0.333	0.8	0.9	2041	1.3E-07
2013	Azizgolshani	Azizgolshani, 2013	Closed	Frequency, degree of cell seeding	5	0.40	0.010	0.40	1–4	2	0.75	0.00001	0.025	3.2	3.6	32	0.14
2019	Li	Li et al., 2019	Closed	Frequency, stimulation voltage, buckle vs. unbuckle	3	0.43	0.028	0.01	1–4	4	0.15	0.00020	0.064	0.8	0.9	13	0.09

*Properties of elastic element provided/estimated from the cited study: L, length; ID, inner diameter; h, wall thickness; E, Young modulus; F_range_, range of tested compression frequencies; F_max_, estimated frequency at which the observed flowrate was maximal; w, width of pincher; Q_max_, maximal flowrate observed in the study. Calculated based on Eq.1&3: h/D, ratio between the wall thickness and the inner diameter; c, estimated pulse wave velocity; c’, with Poisson’s ratio correction for wall thickness; F_n_, estimated natural frequency based on c; Q_prst_, estimated flowrate by a comparable peristaltic pump.*

## Resonant Frequencies

### Natural Frequency Concept

**FIGURE 2:**
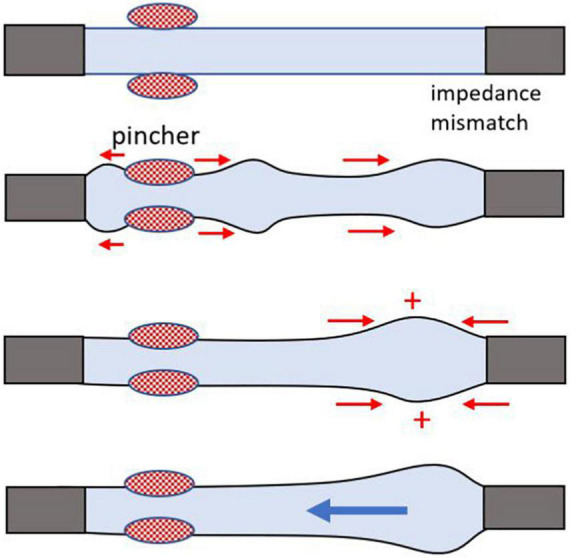
Cartoon illustrating mismatch of impedance that creates a wave reflection site, leading to a one-way flow. A general cartoon of Liebau’s principle for an open tube system. Rhythmic compressions of the flexible tube in the middle do not generate flow, while asymmetrically positioned pinch sites yield a one-way flow. The mismatch of impedance at the junction of the two tubes with different wall compliance creates a wave reflection site, a necessary condition to achieve pumping. Flow direction depends on the compression frequency, geometry, and physical properties of the tubes.

Based on the currently prevailing view, the main mechanism as to how the Liebau pump generates flow is depicted in Figure 2. Pressure waves generated by the periodic compression of the compliant segment reach the points of impedance mismatch. When the pincher is positioned asymmetrically, it leads to a dynamic pressure difference between the two ends of the compliant tube, which in turn generates the flow. It has been argued that for such a difference to be maximal, the system has to be near its resonant frequency (Hickerson et al., 2005; Hickerson and Gharib, 2006). Yet many experimental studies, including our own work (Davtyan and Sarvazyan, 2021), observed significant flow rates at frequencies that are far below the estimated natural frequency (**F**_n_) of the compliant segment. Let us consider the concept of **F**_n_ in more detail.

Frequencies at which the system resonates can be determined experimentally upon hitting the tube with a substantial force and applying a fast Fourier transform analysis to pressure signals (Manopoulos et al., 2020). There is also a simple formula to calculate the natural frequency of a tube:


(1) $$F_{n}\;=\;c/2\text{L}$$

where **L** is the length of a tube and **c** is the velocity of the pressure or pulse wave. The latter can be measured experimentally by tracking the speed of wall deformation or via distally positioned pressure transducers. For thin-walled tubes, pulse wave velocity can be estimated using the Moens-Korteweg equation, where **E** is the Young modulus of the wall, **h** is wall thickness, **D** is the internal diameter, and ρ is fluid density:


(2) $$c\;=\;{\left(Eh/\rho D\right)}^{1/2}$$

For thick-walled vessels, the equation can be further modified using Poisson’s ratio **υ:**


(3) $$c^{\prime }\;=\;{\left(Eh\text{/}\rho D(1-\upsilon^{2})\right)}^{1/2}$$

Most biological materials can be considered incompressible with Poisson’s ratio close to 0.5. Therefore, correcting for Poisson’s ratio basically increases the estimated pulse velocity values by ∼15%.

***Equation 1*** implies that **F**_n_ is inversely proportional to the length of the compliant segment. Therefore, the smaller Liebau pump is, the higher **F**_n_ it is expected to have. Moreover, **F**_n_ is likely to increase as a result of increased values of **c**. This is because wall thickness **h** cannot decrease at the same rate as vessel diameter since this will render vessel too fragile to handle and/or to repetitively compress. Therefore, for very small Liebau pumps, one should expect **h/D** ratio in ***Equation 2*** to increase, leading to a further increase in **c** and consequently in **F**_n_ as per ***Equation 1***.

Calculated **F**_n_ value is affected by the energy losses in the system and is referred to as “damped natural frequency.” These energy losses can be linked to the mass and viscosity of the moving fluid and expanding vessel walls (Babbs, 2010). They also include frictional losses from the interaction of flowing fluid with the walls of the vessel. More friction will occur in vessels with smaller diameters, with more **F**_n_ damping expected in narrow vessels. Additional changes to the calculated **F**_n_ of the complaint segment of the Liebau pump are imposed by the differences in compliance between the soft and stiff tube segments as well as the length of the latter. In the case of a closed flow loop, when the difference between the Young modules of compliant and stiff tubing decreases, the latter becomes part of the resonant system (Meier, 2011).

For the surveyed experimental studies of the Liebau pump listed in Table 2, we calculated pulse wave velocities **c** and undamped **F**_n_ values. This enabled us to compare **F**_n_ with the frequency at which the experimentally recorded flow rate was maximal (**F**_max_). Notably, here **F**_max_ is simply a frequency for which maximal mean flow rate was observed in *each specific study* and not what would have been observed if the *entire* range of frequencies had been examined. For calculations of **F**_n_ we used vessel dimensions and Young modulus provided by the authors, while filling any gaps by using information available elsewhere (indicated by asterisks). As Table 2 shows, with few exceptions, most studies tested frequency ranges that lie *below* calculated undamped **F**_n_. In a few studies that examined frequency ranges that included the predicted **F**_n_, the **F**_max_ was actually very close to the calculated **F**_n_.

The overall conclusion from this section is the following: while the maximal flowrates created by Liebau pumps are predicted to occur near **F**_n_, published experimental studies suggest that sizable flowrates can be created using much lesser frequencies. What we mean by the word “sizable” is considered next.

## Comparison With Peristaltic Pumps of Similar Dimensions

One way to evaluate the performance of Liebau pumps across different experimental studies is to compare them to peristaltic pumps of the same dimensions operating at the same frequency. To find a flow rate by a comparable peristaltic pump, **F**_max_ was multiplied by the volume of displaced fluid, the latter being a product of pincher width (**w**) and cross-sectional area of the tube (**A**):


(4) Q=pFwAmax

**FIGURE 3:**
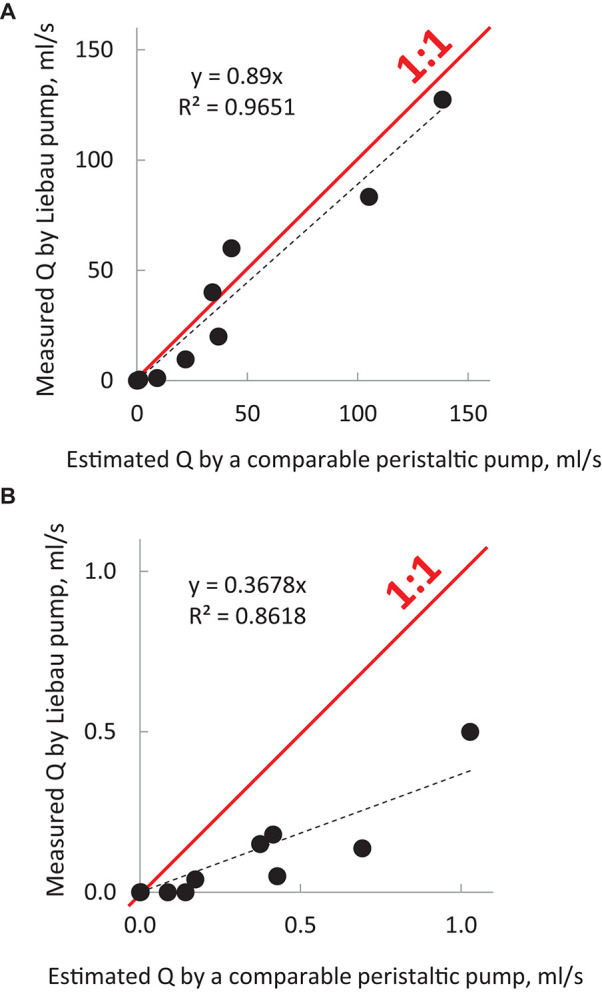
Output of Liebau pumps in comparison to peristaltic pumps of the same dimensions. The graphs were compiled using MS Excel from published studies listed in Table 2. They illustrate the relative efficiency of Liebau pumps compared to peristaltic pumps of the same dimensions and compression frequencies. Dots above the red line indicate flow rates that were higher than the ones created by the peristaltic pumps; below the red line are lower. For macroscopic pumps, flow created by Liebau pumps was ∼90% from that of peristaltic flow, while for smaller pumps it was ∼40%. Data also point to a significant correlation between the two ways to pump fluid. To calculate the output of a comparable peristaltic pump, the cross-sectional area of the tube was multiplied by the width of the pincher element and the compression frequency at which the maximal flow was observed in each cited study of the Liebau pump. Red rectangle in **(A)** encompasses datapoints for micropumps. The latter has been shown in **(B)** using different scales.

Note that to calculate the expected flow rate by peristaltic pumps, we assumed a complete closure of the lumen. The same cannot be expected for all Liebau pumps listed in Table 2, since the degree of lumen occlusion varied between the studies (the latter value is often omitted, with only ∼30% of surveyed studies mentioning it). Therefore, the reported maximal flow rates created by the Liebau pumps could have been higher if a complete lumen closure had been achieved. The outcome of the comparison is shown graphically in Figure 3 using values listed in Table 2. The Figure 3A shows data from all surveyed experimental studies, while Figure 3B zooms in on pumps with the smallest dimensions. These plots suggest two things. First, there is a high degree of correlation between the maximal flow rates achievable by peristaltic vs. Liebau-based pumps of the same dimensions (*R*^2^ = 0.97). Second, for larger pumps, this relationship is approaching 1:1, with two studies (Hickerson et al., 2005; Bringley et al., 2008) reporting flow rates exceeding the estimated peristaltic-based flow. The latter is rather remarkable since in the case of the peristaltic pump it is the entire length of the tube that needs to be engaged in active contraction, while in the Liebau pump it is only a small segment of the tube. In fact, when averaged across surveyed experimental studies, the width of the pincher was only about 12 ± 6% of the total length of the tube.

## Flow Rate-Frequency Relationship

Nearly all studies of Liebau pumps report pulsatile flow, the presence of flow reversals, and a non-linear flow rate-frequency relationship. Yet the exact shape of the latter curve, as well as the number of flow-frequency peaks, varies dramatically across studies. In addition, while some report maximal flowrate near **F**_n_ (Hickerson et al., 2005; Kozlovsky et al., 2015; Manopoulos et al., 2020), others report it being close to zero (Takagi and Takahashi, 1985; Borzì and Propst, 2003; Meier, 2011). In the latter cases, the performance of the valveless pump is maximum on either side of the resonant frequency, passing through zero flow rate at the resonant frequency. An interesting suggestion as to why such different patterns have been observed was made by Wen and Chang (2009). They have shown that the flow-frequency relationship shifts when the resistances between the two sides of stiff tubing to which the compliant tube is connected are different. Whether unintended small variations in resistances between the two sides can explain differently shaped frequency-flow rate curves near **F**_n_ values awaits further exploration.

## Flow Velocity Profiles and Womersley Number

To scale and characterize the dynamics of flow in differently sized vessels, dimensionless Reynolds and Womersley numbers are commonly used. For cylindrical vessels under a steady pressure gradient, the Reynolds number<2,000 predicts laminar flow with a parabolic velocity profile. The Womersley number is used to characterize the velocity profile during pulsatile pressure conditions. The Womersley number is calculated using the values of fluid density ρ, dynamic viscosity μ, angular frequency ω, and the vessel radius **R**:


(5) $$W_{o}{}_{=}R{\left(\rho \omega /\mu \right)}^{1/2}$$

When **W**_o_ < 1, the flow tracks the oscillating pressure gradient, and the velocity profiles exhibit a parabolic shape. When **W**_o_ > 10, the velocity profile is flat or plug-like, and the flow is phase-shifted relative to the oscillating pressure gradient. Values of 1 < **W**_o_ < 10 represents intermediate regimes (Manopoulos et al., 2020). For the surveyed non-biological studies of Liebau pumps listed under Table 2, typical **W**_o_ values exceed 10. In contrast, the two experimental attempts at creating BLPs (Azizgolshani, 2013; Li et al., 2019) described under see section “Published Attempts to Create Functional Biofabricated Liebau Pumps” had **W**_o_ < 10. This was due to a combination of low frequency and small vessel diameters. In cases when solutions that mimic blood viscosity were used, the **W**_o_ values further declined. This was the case in our own experiments that tested the performance of the Liebau pump with physiologically relevant vessel dimensions, viscosity, and compression frequencies (Davtyan and Sarvazyan, 2021).

The Womersley number plays a role in calculating the mean flow rate **Q** from particle tracking, the latter method being one of the easiest ways to estimate the flow experimentally:


(6) Q=πRK2vw

where **R** is the radius of the cylindrical vessel and **v** is the maximal linear velocity of the particles flowing midstream. Coefficient **K**_w_ enables one to account for different velocity profiles. It is equal to 0.5 when **Wo < 1** and the velocity profile is parabolic. When **Wo > 10**, the **K**_w_ becomes close to 1, since when flow is plug-like, the velocity profile of flowing particles is nearly the same across the entire volume of the tube. For intermediate regimes when **1 < W_o_ < 10** an empirically derived formula can be used to derive coefficient **K_w_.** This formula was developed by Ponzini et al. (2010) and is included in Supplementary Excel File.

## Choice of Vessel Material to Build Biofabricated Liebau Pumps *ex vivo*

To get closer to **F**_n_ within the physiological range of frequencies, i.e., 1–4 Hz, it is best to select highly stretchable materials with low Young modulus while having high tensile strength to prevent breakage. There are two possible approaches. The first approach is to excise tubular structures from an organism of choice and use them in their native, processed, or decellularized form. The most obvious choice appears to be pieces of arteries or veins. However, in their natural form, the values of Young modulus for blood vessels are still rather high. When stretched transversally, reported values of Young modulus for blood vessels span from 0.5 to 5 MPa (Riley et al., 1992; Egorov et al., 2008). When non-destructive decellularization protocols are being used, the elastic modulus of vessels changes very little (Daniel et al., 2005; Crapo et al., 2011). This is because the mechanical properties of vessels are mainly determined by interwoven collagen and elastin fibers. Other tubular structures that can be excised and used to create a compliant segment for BLPs include intestine (Herbert et al., 1993), ureter (Narita et al., 2008), or their components such as small-intestine submucosa, for example (Roeder et al., 1999). One can also consider additional treatments that can lower **E** values of these vessels while retaining their integrity, mild enzymatic treatment being one example (Trabelsi et al., 2020).

The second approach to creating BLP-compliant segments is to use the ever-expanding arsenal of biofabrication tools (Pashneh-Tala et al., 2016). Suitable tubular scaffolds can be created using casting, 3D printing, electrospinning, weaving, or other techniques (Chang and Niklason, 2017; Elomaa and Yang, 2017; Song et al., 2018). The range of biocompatible materials that can be used is vast, and they can be mixed or modified to achieve the desired stiffness (Bello et al., 2020). The Young modulus of unenforced simple hydrogels, such as gelatin, acrylamide, or agarose, is on the order of a few kPA (Chen et al., 2018; Lee et al., 2019). It can be enhanced by several orders of magnitude by including different additives (Le Goff et al., 2015; Yan et al., 2017). Collagen, elastin, and other extracellular matrix components are other obvious choices. Although pure collagen fibers are very stiff, with **E** reaching 1.2 GPa, the strength of structures made from collagen fibers can be adjusted by changing protein concentration, type, crosslinking, and inclusion of other ECM components (Gosline et al., 2002).

## Creation of Biofabricated Liebau Pumps *in vivo*

The above-described strategies to approach **F**_n_ (i.e., by decreasing elastic modulus and **h/D** ratio, while increasing vessel length **L**) can work to create and test *ex vivo* BLPs. But what about creating BLPs *in vivo* by wrapping tissue engineered muscle around a vessel of choice? For medium-sized blood vessels, the **h/D** ratio is about 0.3 for arteries and 0.1 for veins. The lowest reported values of Young modulus for collagen-rich arteries and veins are about 0.2–0.5 MPa (Daniel et al., 2005). Therefore, if a 10 cm piece of vein with a diameter of 1 cm and an **E** of 0.2 MPa is used as a compliant segment of the Liebau pump, the Moens-Korteweg equation yields c = ∼4 m/s and **F**_n_ = 22 Hz (see Supplementary Appendix for formulas). The later value is too high for a pincher made from muscle cells. For smaller vessels, the **F**_n_ will be even higher. And if one estimates **F**_n_ values for an embryonic heart tube of a zebrafish by eyeballing its dimensions [200, 60, 20 μm for **L**, **D,** and **h,** respectively (Forouhar et al., 2006)], while assuming the lowest possible **E** of 2 kPA, the **F**_n_ will be > 2000 Hz, which is an impossible range of frequencies for biological pumps.

The above estimates of **F**_n_ seems to question the feasibility of creating *in vivo* BLPs while using a muscle ring as a pincher element. However, this is far from the case. Data shown in Figure 3B illustrate that despite being less efficient than their macro versions (i.e., Figure 3A), micro Liebau pumps working far from their **F**_n_ values generate flows that are about 10–30% of their peristaltic equivalents. Also, several modifications of Liebau pumps have been suggested. These modifications resemble features of embryonic hearts and parts of circulation in lower invertebrates. They can help to increase flow rates at frequencies lower than **F**_n_ and are considered next.

## Modifications to Increase Biofabricated Liebau Pump Output

Previous paragraphs considered the simplest configuration of Liebau pumps that consisted of a single-walled cylindrical compliant segment with a single compression element. As we argued above, in this basic configuration BLPs with the length ranging from millimeters to several centimeters will yield miniscule flowrates when compressed with physiological frequencies. By mimicking scenarios that can be encountered *in vivo*, one can try to modify the basic configuration of the Liebau pump to increase the flow. One study, for example, documented the beneficial effect of kinks by showing that by bending compliant tubes in several places, one can significantly increase its performance (Hiermeier and Männer, 2017). Another way to increase the mean flow rate was suggested by a theoretical study by Loumes et al. (2008). The authors tried to mimic the effects of cardiac jelly, which is present during the early stages of embryonic heart development. The proposed model predicted that the addition of a thick gelatinous layer to the inner wall of the complaint segment would greatly increase the amplitude of the pressure waves and increase the mean flow rates of the pump. Notably, in the presence of such a gelatinous layer, the vessel’s lumen can be constricted to a larger degree using the same degree of outer layer shortening. The letter is particularly important for a pincher element made of striated muscle, which can only shorten to a ∼15% of its initial length. Another interesting approach to better mimic putative Liebau pumps was to add cavities before and after the pincher element (Kozlovsky et al., 2015). This was again a purely computational study, conclusions from which await experimental proof. An additional way to increase the force by which muscular band compresses the compliant tube can be to mimic the spiral structure of the cardiac wall by creating a pincher using multiple bands with alternating directions of the fibers (Torrent-Guasp et al., 2001).

**FIGURE 4:**
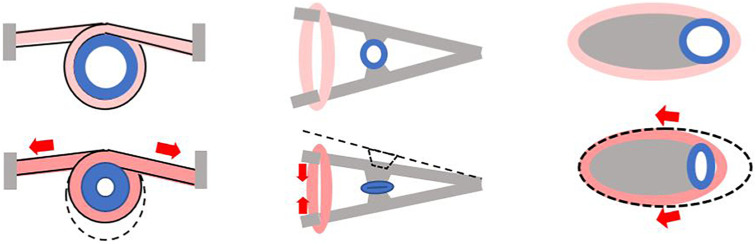
Alternative ways to create a pincher from tissue engineered muscle. Gray color depicts stationary elements that can be created from bone-like or similar material. Pink color indicates tissue engineered muscle. Cross section of the compliant segment of Liebau pump is shown in blue. Each panel shows a pincher before (top) and during (bottom) contraction with red arrows depicting direction of muscle shortening.

One can also suggest building a Liebau pump using biological elements but without mimicking *in vivo* scenarios. This can be done by changing the position of the compression element relative to the vessel. Different configurations that can help to increase the degree of lumen compression can be envisioned (Figure 4). Although such configurations are unlikely to be considered in lieu of clinical treatments, one can imagine using them for organ-on-a-chip or similar applications.

## Relative Efficiency of Liebau Pumps

Flow and pressure outputs of Liebau pumps are highly non-linear and depend on multiple factors, including those listed in Tables 1, 2. Therefore, it is rather difficult to quantitively compare their energy efficiency to other types of pumping devices. As a rough estimate one can use a relationship between the maximal flowrate and the pump dimensions, an approach taken by Laser and Santiago to create a chart comparing a wide range of micropumps, including piezoelectric, thermopneumatic, electrostatic and electroosmotic devices (Laser and Santiago, 2004). The experimental data from Table 2 and as well as evaluation by Meier (2011), places Liebau pumps on the top of that chart. Data shown Figure 3 also suggest that at their optimal frequencies the output of Liebau pumps, particularly those on a centimeter scale, is comparable to their peristaltic counterparts. Interestingly, a significant increase in the flowrate can be achieved by the inclusion of the valves into the Liebau pump circuit (Moser et al., 1998). From the viewpoint of future clinical implementation though, addition of the valves makes little sense since their absence is probably the most beneficial feature of the Liebau pump design.

## Insights From the First Biofabricated Liebau Pump Attempt

Although the first published BLP attempt (Li et al., 2019) using tissue engineered rings of skeletal muscle around hydrogel tubes yielded negligible flow rates, it enabled the authors to make several interesting observations. Notably, such insights were never brought up by studies that built Liebau pumps from non-biological components or relied on simulations. Indeed, contraction of a ring of muscle cells wrapped around a vessel does not work like a mechanical pincher that compresses vessels in one dimension. Instead, the authors observed that in many cases, any significant contraction of the muscular ring led to an inward deformation of the vessel wall. Such “buckling” then impacted the calculation of a new radius of the tube as well as the degree of lumen compression. Also, the authors revealed that buckling had an unexpected effect on the flow rate-frequency relationship. When the tube was buckled, there was less resistance to the act of compression, so the degree of muscle shortening was higher at the same degree of electrical stimulation. Moreover, when stimulation frequency increased with electrical pulses arriving during relaxation, contraction amplitude decreased, giving rise to frequency dependence of contraction amplitude, i.e., the higher was the frequency, the lower was the amplitude of contraction. The authors argued that for the unbuckled case, restoring force of the elastic tube was high compared with the buckled tube, and contraction amplitude was less sensitive to the contraction frequency.

New insights, such as above, are expected from future attempts to build Liebau pumps using biological materials. We hope that this publication will encourage others to explore this fascinating phenomenon and stimulate additional experimental efforts to build valveless impedance pumps using biological materials.

## Conclusion

Cumulative evidence from different labs point to physiological feasibility of Liebau-based valveless pumping warranting efforts to create such pumps using tissue engineering and other biofabrication tools. Such efforts will provide additional insights into performance of these pumps *in vivo* as well as further our understanding of the fundamental mechanisms driving blood flow during early development. Liebau-based biomimetic pumps can also serve as energy-efficient flow generators in organ-on-a-chip devices or be implanted to assist flow during various disease states.

## Author Contributions

NS conceived and wrote the manuscript.

## Conflict of Interest

The author declares that the research was conducted in the absence of any commercial or financial relationships that could be construed as a potential conflict of interest.

## Publisher’s Note

All claims expressed in this article are solely those of the authors and do not necessarily represent those of their affiliated organizations, or those of the publisher, the editors and the reviewers. Any product that may be evaluated in this article, or claim that may be made by its manufacturer, is not guaranteed or endorsed by the publisher.

## References

[B1] Anderson R. H. (1981). Hearts and Heart-like Organs. Volume 1. Comparative anatomy and development. *J. Anat.* 133(Pt 1) 104.

[B2] Auerbach D. Moehring W. Moser M. (2004). An analytic approach to the Liebau problem of valveless pumping. *Cardiovasc. Eng.* 4 201–207. 10.1023/b:care.0000031549.13354.5e

[B3] Azizgolshani H. (2013). *Tissue Engineering Active Biological Machines: Bio-Inspired Design, Directed Self-Assembly, and Characterization of Muscular Pumps Simulating the Embryonic Heart.* Ph.D. Thesis. California, CA: California Institute of Technology.

[B4] Babbs C. F. (2010). Behavior of a viscoelastic valveless pump: a simple theory with experimental validation. *Biomed. Eng. Online* 9:42. 10.1186/1475-925X-9-42 20807440 PMC2944263

[B5] Bello A. B. Kim D. Kim D. Park H. Lee S. H. (2020). Engineering and functionalization of gelatin biomaterials: from cell culture to medical applications. *Tissue Eng. Part B Rev.* 26 164–180. 10.1089/ten.TEB.2019.0256 31910095

[B6] Bers D. (2001). *Excitation-Contraction Coupling and Cardiac Contractile Force*, 2nd Edn. Berlin: Springer Science & Business Media.

[B7] Borzì A. Propst G. (2003). Numerical investigation of the Liebau phenomenon. *Zeitschrift Angew Math. Phys.* 54 1050–1072.

[B8] Breckwoldt K. Letuffe-Brenière D. Mannhardt I. Schulze T. Ulmer B. Werner T. (2017). Differentiation of cardiomyocytes and generation of human engineered heart tissue. *Nat. Protoc.* 12 1177–1197. 28492526 10.1038/nprot.2017.033

[B9] Bringley T. T. Chilress S. Vandenberghe N. Zhang J. (2008). An experimental investigation and a simple model of a valveless pump. *Phys. Fluids* 20:33602.

[B10] Burton R. A. B. Klimas A. Ambrosi C. M. Tomek J. Corbett A. Entcheva E. (2015). Optical control of excitation waves in cardiac tissue. *Nat. Photonics* 9 813–816. 10.1038/nphoton.2015.196 27057206 PMC4821438

[B11] Carrier R. L. Papadaki M. Rupnick M. Schoen F. J. Bursac N. Langer R. (1999). Cardiac tissue engineering: cell seeding, cultivation parameters, and tissue construct characterization. *Biotechnol. Bioeng.* 64 580–589. 10.1002/(sici)1097-0290(19990905)64:5<580::aid-bit8>3.0.co;2-x 10404238

[B12] Chang W. G. Niklason L. E. (2017). A short discourse on vascular tissue engineering. *NPJ Regen. Med.* 2:7. 10.1038/s41536-017-0011-6 29057097 PMC5649630

[B13] Chen R. Xu X. Yu D. Xiao C. Liu M. Huang J. (2018). Highly stretchable and fatigue resistant hydrogels with low Young’s modulus as transparent and flexible strain sensors. *J. Mater. Chem. C. R. Soc. Chem.* 6:11193. 10.1039/c8tc02583e

[B14] Crapo P. M. Gilbert T. W. Badylak S. F. (2011). An overview of tissue and whole organ decellularization processes. *Biomaterials* 32 3233–3243. 10.1016/j.biomaterials.2011.01.057 21296410 PMC3084613

[B15] Cvetkovic C. Raman R. Chan V. Williams B. J. Tolish M. Bajaj P. (2014). Three-dimensionally printed biological machines powered by skeletal muscle. *Proc. Natl. Acad. Sci. U.S.A.* 111 10125–10130. 10.1073/pnas.1401577111 24982152 PMC4104884

[B16] Dai W. Hale S. L. Kloner R. A. (2006). Cardiac cells implanted within the outer aortic wall of rats generate measurable contractile force. *Regen. Med.* 1 119–124. 10.2217/17460751.1.1.119 17465826

[B17] Daniel J. Abe K. McFetridge P. S. (2005). Development of the human umbilical vein scaffold for cardiovascular tissue engineering applications. *ASAIO J.* 51 252–261. 10.1097/01.mat.0000160872.41871.7e 15968956

[B18] Davtyan R. Sarvazyan N. A. (2021). Output of a valveless Liebau pump with biologically relevant vessel properties and compression frequencies. *Sci. Rep.* 11:11505. 10.1038/s41598-021-90820-4 34075100 PMC8169938

[B19] Egorov V. Tsyuryupa S. Kanilo S. Kogit M. Sarvazyan A. (2008). Soft tissue elastometer. *Med. Eng. Phys.* 30 206–212. 10.1016/j.medengphy.2007.02.007 17383214 PMC2581446

[B20] Elomaa L. Yang Y. P. (2017). Additive manufacturing of vascular grafts and vascularized tissue constructs. *Tissue Eng. Part B Rev.* 23 436–450. 10.1089/ten.TEB.2016.0348 27981886 PMC5652978

[B21] Entcheva E. (2013). Cardiac optogenetics. *Am. J. Physiol. Heart Circ. Physiol.* 304 H1179–H1191. 23457014 10.1152/ajpheart.00432.2012 PMC3652095

[B22] Entcheva E. Kay M. W. (2020). Cardiac optogenetics: a decade of enlightenment. *Nat. Rev. Cardiol.* 18 349–367. 10.1038/s41569-020-00478-0 33340010 PMC8127952

[B23] Forouhar A. S. Liebling M. Hickerson A. Nasiraei-Moghaddam A. Tsai H. J. Hove J. R. (2006). The embryonic vertebrate heart tube is a dynamic suction pump. *Science* 312 751–753. 10.1126/science.1123775 16675702

[B24] Gosline J. Lillie M. Carrington E. Guerette P. Ortlepp C. Savage K. (2002). Elastic proteins: biological roles and mechanical properties. *Philos. Trans. R Soc. B Biol. Sci.* 357 121–132. 10.1098/rstb.2001.1022 11911769 PMC1692928

[B25] Herbert S. T. Badylak S. F. Geddes L. A. Hillberry B. Lantz G. C. Kokini K. (1993). Elastic modulus of prepared canine jejunum, a new vascular graft material. *Ann. Biomed. Eng.* 21 727–733. 10.1007/BF02368651 8116923

[B26] Hickerson A. I. Gharib M. (2006). On the resonance of a pliant tube as a mechanism for valveless pumping. *J. Fluid. Mech.* 555 141–148. 10.1017/s0022112006009220

[B27] Hickerson A. I. Rinderknecht D. Gharib M. (2005). Experimental study of the behavior of a valveless impedance pump. *Exp. Fluids* 38 534–540. 10.1007/s00348-005-0946-z

[B28] Hiermeier F. Männer J. (2017). Kinking and torsion can significantly improve the efficiency of valveless pumping in periodically compressed tubular conduits. Implications for understanding of the form-function relationship of embryonic heart tubes. *J. Cardiovasc. Dev. Dis.* 4:19. 10.3390/jcdd4040019 29367548 PMC5753120

[B29] Jaffrin M. Y. Shapiro A. H. (1971). Peristaltic pumping. *Annu. Rev. Fluid Mech.* 3 13–37.

[B30] Johansen K. Burggren W. Bourne G. H. (1980). “Cardiovascular function in the lower vertebrates,” in *Hear Hear Organs*, ed. Bourne G. H. (London: Academic Press), 61–117. 10.1016/b978-0-12-119401-7.50009-8

[B31] Juhas M. Engelmayr G. C. Fontanella A. N. Palmer G. M. Bursac N. (2014). Biomimetic engineered muscle with capacity for vascular integration and functional maturation in vivo. *Proc. Natl. Acad. Sci. U.S.A.* 111 5508–5513. 10.1073/pnas.1402723111 24706792 PMC3992675

[B32] Jung E. (2007). A mathematical model of valveless pumping: a lumped model with time-dependent compliance, resistance, and inertia. *Bull. Math. Biol.* 69 2181–2198. 10.1007/s11538-007-9208-y 17457651

[B33] Jung E. Peskin C. S. (2002). Two-dimensional simulations of valveless pumping using the immersed boundary method. *SIAM J. Sci. Comput.* 23 19–45. 10.1007/s12013-011-9157-9 21336589

[B34] Kang H. W. Lee S. J. Ko I. K. Kengla C. Yoo J. J. Atala A. (2016). A 3D bioprinting system to produce human-scale tissue constructs with structural integrity. *Nat. Biotechnol.* 34 312–319. 10.1038/nbt.3413 26878319

[B35] Karabekian Z. Ding H. Stybayeva G. Ivanova I. Muselimyan N. Haque A. (2015a). HLA Class I depleted hESC as a source of hypoimmunogenic cells for tissue engineering applications. *Tissue Eng. Part A* 21 2559–2571. 10.1089/ten.TEA.2015.0105 26218149 PMC4605353

[B36] Karabekian Z. Idress S. Jamshidi A. Posnack N. G. Sarvazyan N. A. Idrees S. (2015b). Downregulation of beta-microglobulin to diminish T-lymphocyte lysis of non-syngeneic cell sources of engineered heart tissue constructs. *Biomed. Mater.* 10:34101. 10.1088/1748-6041/10/3/034101 25775354 PMC8083821

[B37] Khodabukus A. Madden L. Prabhu N. K. Koves T. R. Jackman C. P. Muoio D. M. (2019). Electrical stimulation increases hypertrophy and metabolic flux in tissue-engineered human skeletal muscle. *Biomaterials* 198 259–269. 10.1016/j.biomaterials.2018.08.058 30180985 PMC6395553

[B38] Koti P. Muselimyan N. Mirdamadi E. Asfour H. Sarvazyan N. A. (2019). Use of GelMA for 3D printing of cardiac myocytes and fibroblasts. *J. 3D Print Med.* 3 11–22. 10.2217/3dp-2018-0017 31555480 PMC6760315

[B39] Kozlovsky P. Bryson-Richardson R. J. Jaffa A. J. Rosenfeld M. Elad D. (2016). The driving mechanism for unidirectional blood flow in the tubular embryonic heart. *Ann. Biomed. Eng. Springer N. Y. LLC* 44:3069. 10.1007/s10439-016-1620-8 27112782

[B40] Kozlovsky P. Rosenfeld M. Jaffa A. J. Elad D. (2015). Dimensionless analysis of valveless pumping in a thick-wall elastic tube: application to the tubular embryonic heart. *J. Biomech.* 48:1652. 10.1016/j.jbiomech.2015.03.001 25835790

[B41] Laser D. J. Santiago J. G. (2004). A review of micropumps. *J. Micromech. Microeng.* 14:R35.

[B42] Le Goff K. J. Gaillard C. Helbert W. Garnier C. Aubry T. (2015). Rheological study of reinforcement of agarose hydrogels by cellulose nanowhiskers. *Carbohydr. Polym.* 116 117–123. 10.1016/j.carbpol.2014.04.085 25458280

[B43] Lee D. Zhang H. Ryu S. (2019). “Elastic modulus measurement of hydrogels,” in *Cellulose-Based Superabsorbent Hydrogels. Polymers and Polymeric Composites: A Reference Series*, ed. Mondal M. (Cham: Springer), 865–884. 10.1007/978-3-319-77830-3_60

[B44] Lee V. C. C. Abakr Y. A. Woo K. C. (2013). Valveless pumping using a two-stage impedance pump. *Front. Mech. Eng.* 8:311. 10.1007/s11465-013-0270-x

[B45] Lee V. C. C. Chai C. H. Law M. C. Wee S. K. (2017). On the analysis of impedance-driven reverse flow dynamics. *J. Eng. Sci. Technol.* 12 451–459.

[B46] Li Z. Seo Y. Aydin O. Elhebeary M. Kamm R. D. Kong H. (2019). Biohybrid valveless pump-bot powered by engineered skeletal muscle. *Proc. Natl. Acad. Sci. U.S.A.* 116 1543–1548. 10.1073/pnas.1817682116 30635415 PMC6358718

[B47] Liebau G. (1954). Uber ein ventilloses Pumpprinzip. *Naturwissenschaften* 41:327. 10.1007/bf00644490 5736280

[B48] Liebau G. (1955). Die Strömungsprinzipien des Herzens. *Z. Kreislaufforsch.* 44:677. 13291885

[B49] Longatti P. (2018). The Liebau phenomenon: a translational approach to new paradigms of CSF circulation and related flow disturbances. *Child’s Nerv. Syst.* 34 227–233. 10.1007/s00381-017-3653-1 29124390

[B50] Loumes L. Avrahami I. Gharib M. (2008). Resonant pumping in a multilayer impedance pump. *Phys. Fluids* 20:23013.

[B51] Ma C. Peng Y. Li H. Chen W. (2021). Organ-on-a-Chip: a new paradigm for drug development. *Trends Pharmacol. Sci.* 42 119–133. 10.1016/j.tips.2020.11.009 33341248 PMC7990030

[B52] Männer J. Wessel A. Yelbuz T. M. (2010). How does the tubular embryonic heart work? Looking for the physical mechanism generating unidirectional blood flow in the valveless embryonic heart tube. *Dev. Dyn.* 239 1035–1046. 10.1002/dvdy.22265 20235196

[B53] Manopoulos C. Tsangaris S. Mathioulakis D. (2020). Net flow generation in closed-loop valveless pumping. *Proc. Inst. Mech. Eng. Part C J. Mech. Eng. Sci.* 234:2126. 10.1177/0954406220904110

[B54] Manopoulos C. G. Mathioulakis D. S. Tsangaris S. G. (2006). One-dimensional model of valveless pumping in a closed loop and a numerical solution. *Phys. Fluids* 18:17106.

[B55] Meier J. (2011). *A Novel Experimental Study of A Valveless Impedance Pump for Applications at Lab-On-Chip, Microfluidic, and Biomedical Device Size Scales.* Ph.D. Thesis. California, CA: California Institute of Technology.

[B56] Moser M. Huang J. Schwarz G. Kenner T. Noordergraaf A. (1998). Impedance defined flow. Generalisation of William Harvey’s concept of the circulation—370 years later. *Int. J. Cardiovasc. Med. Sci.* 1 205–211.

[B57] Narita Y. Kagami H. Matsunuma H. Murase Y. Ueda M. Ueda Y. (2008). Decellularized ureter for tissue-engineered small-caliber vascular graft. *J. Artif. Organs* 11 91–99. 10.1007/s10047-008-0407-6 18604613

[B58] Nguyen T.-U. Bashur C. A. Kishore V. (2016). Impact of elastin incorporation into electrochemically aligned collagen fibers on mechanical properties and smooth muscle cell phenotype. *Biomed. Mater.* 11:25008. 10.1088/1748-6041/11/2/025008 26987364

[B59] Nunes S. S. Miklas J. W. Liu J. Aschar-Sobbi R. Xiao Y. Zhang B. (2013). Biowire: a platform for maturation of human pluripotent stem cell-derived cardiomyocytes. *Nat. Methods* 10 781–787. 23793239 10.1038/nmeth.2524 PMC4071061

[B60] Ottesen J. T. (2003). Valveless pumping in a fluid-filled closed elastic tube-system: one-dimensional theory with experimental validation. *J. Math. Biol.* 46 309–332. 10.1007/s00285-002-0179-1 12673509

[B61] Pahlevan N. M. Gharib M. (2013). In-vitro investigation of a potential wave pumping effect in human aorta. *J. Biomech.* 46 2122–2129. 10.1016/j.jbiomech.2013.07.006 23915578

[B62] Pashneh-Tala S. MacNeil S. Claeyssens F. (2016). The tissue-engineered vascular graft - Past, present, and future. *Tissue Eng. Part B Rev.* 22 68–100. 10.1089/ten.teb.2015.0100 26447530 PMC4753638

[B63] Ponzini R. Vergara C. Rizzo G. Veneziani A. Roghi A. Vanzulli A. (2010). Womersley number-based estimates of blood flow rate in doppler analysis: in vivo validation by means of phase-contrast MRI. *IEEE Trans. Biomed. Eng.* 57 1807–1815. 10.1109/TBME.2010.2046484 20659826

[B64] Radisic M. Park H. Shing H. Consi T. Schoen F. J. Langer R. (2004). Functional assembly of engineered myocardium by electrical stimulation of cardiac myocytes cultured on scaffolds. *Proc. Natl. Acad. Sci. U.S.A.* 101 18129–18134. 10.1073/pnas.0407817101 15604141 PMC539727

[B65] Raman R. Cvetkovic C. Uzel S. G. M. Platt R. J. Sengupta P. Kamm R. D. (2016). Optogenetic skeletal muscle-powered adaptive biological machines. *Proc. Natl. Acad. Sci. U.S.A.* 113 3497–3502. 10.1073/pnas.1516139113 26976577 PMC4822586

[B66] Reeser K. Doiron A. L. (2019). Three-dimensional printing on a rotating cylindrical mandrel: a review of additive-lathe 3D printing technology. 3D Print. *Addit. Manuf. Mary Ann. Liebert Inc.* 6 293–307. 10.1089/3dp.2019.0058

[B67] Riley W. A. Barnes R. W. Evans G. W. Burke G. L. (1992). Ultrasonic measurement of the elastic modulus of the common carotid artery: the atherosclerosis risk in communities (aric) study. *Stroke* 23 952–956. 10.1161/01.str.23.7.952 1615543

[B68] Rinderknecht D. Hickerson A. I. Gharib M. (2005). A valveless micro impedance pump driven by electromagnetic actuation. *J. Micromech. Microeng.* 15:861. 10.1088/0960-1317/15/4/026

[B69] Roeder R. Wolfe J. Lianakis N. Hinson T. Geddes L. A. Obermiller J. (1999). Compliance, elastic modulus, and burst pressure of small-intestine submucosa (SIS), small-diameter vascular grafts. *J. Biomed. Mater. Res.* 47:65. 10.1002/(sici)1097-4636(199910)47:1<65::aid-jbm9>3.0.co;2-f 10400882

[B70] Ronaldson-Bouchard K. Yeager K. Teles D. Chen T. Ma S. Song L. J. (2019). Engineering of human cardiac muscle electromechanically matured to an adult-like phenotype. *Nat. Protoc.* 14 2781–2817. 10.1038/s41596-019-0189-8 31492957 PMC7195192

[B71] Rosenfeld M. Avrahami I. (2010). Net flow rate generation by a multi-pincher impedance pump. *Comput. Fluids* 39:1634. 10.1016/j.compfluid.2010.05.016

[B72] Santhanakrishnan A. Miller L. A. (2011). Fluid dynamics of heart development. *Cell Biochem. Biophys.* 61 1–22. 10.1007/s12013-011-9158-8 21327946

[B73] Santhanakrishnan A. Nguyen N. Cox J. G. Miller L. A. (2009). Flow within models of the vertebrate embryonic heart. *J. Theor. Biol.* 259 449–461. 10.1016/j.jtbi.2009.04.020 19410580

[B74] Sarvazyan N. (2014a). Creating biological pumps using engineered heart tissue. *Tissue Eng. Part A.* 20:S46.

[B75] Sarvazyan N. (2014b). Thinking outside the heart: use of engineered cardiac tissue for treatment of chronic deep venous insufficiency. *J. Cardiovasc. Pharmacol. Ther.* 19 394–401. 10.1177/1074248413520343 24500906 PMC5837029

[B76] Schuurman W. Khristov V. Pot M. W. Van Weeren P. R. Dhert W. J. A. Malda J. (2011). Bioprinting of hybrid tissue constructs with tailorable mechanical properties. *Biofabrication* 3:21001. 10.1088/1758-5082/3/2/021001 21597163

[B77] Song H. H. G. Rumma R. T. Ozaki C. K. Edelman E. R. Chen C. S. (2018). Vascular tissue engineering: progress, challenges, and clinical promise. *Cell Stem Cell* 22 340–354. 10.1016/j.stem.2018.02.009 29499152 PMC5849079

[B78] Swift L. Karimi V. Velez M. Simonyan H. Patev N. Posnack N. G. (2014). Engineered heart tissue to aid venous flow. *Tissue Eng. Part A.* 20 S119.

[B79] Takagi S. Takahashi K. (1985). Study of a piston pump without valves: pumping effect and resonance in a pipe-capacity-system with a t-junction. *Bull. Japan Soc. Mech. Eng.* 28 831–836.

[B80] Tandon N. Cannizzaro C. Chao P.-H. G. Maidhof R. Marsano A. Au H. T. H. (2009). Electrical stimulation systems for cardiac tissue engineering. *Nat. Protoc.* 4 155–173. 10.1038/nprot.2008.183 19180087 PMC2775058

[B81] Thomann H. (1978). A simple pumping mechanism in a valveless tube. *Zeitschrift. Angew Math. Phys. ZAMP.* 29:169.

[B82] Timmermann S. Ottesen J. T. (2009). Novel characteristics of valveless pumping. *Phys. Fluids* 21:53601.

[B83] Torrent-Guasp F. Buckberg G. D. Clemente C. Cox J. L. Coghlan H. C. Gharib M. (2001). The structure and function of the helical heart and its buttress wrapping. I. The normal macroscopic structure of the heart. *Semin. Thorac. Cardiovasc. Surg.* 13 301–319. 11807730 10.1053/stcs.2001.29953

[B84] Trabelsi O. Dumas V. Breysse E. Laroche N. Avril S. (2020). In vitro histomechanical effects of enzymatic degradation in carotid arteries during inflation tests with pulsatile loading. *J. Mech. Behav. Biomed. Mater.* 103:103550. 10.1016/j.jmbbm.2019.103550 32090945

[B85] Wen C. Y. Chang H. T. (2009). Design and characterization of valveless impedance pumps. *J. Mech.* 25:345. 10.1017/s1727719100002835

[B86] Yan X. Chen Q. Zhu L. Chen H. Wei D. Chen F. (2017). High strength and self-healable gelatin/polyacrylamide double network hydrogels. *J. Mater. Chem. B* 5:7683. 10.1039/c7tb01780d 32264369

[B87] Zawieja D. C. (2009). Contractile physiology of lymphatics. *Lymphat Res. Biol.* 7 87–96. 10.1089/lrb.2009.0007 19534632 PMC2925033

[B88] Zhang B. Korolj A. Lai B. F. L. Radisic M. (2018). Advances in organ-on-a-chip engineering. *Nat. Rev. Mater.* 3 257–278.

[B89] Zhang D. Shadrin I. Y. Lam J. Xian H.-Q. Snodgrass H. R. Bursac N. (2013). Tissue-engineered cardiac patch for advanced functional maturation of human ESC-derived cardiomyocytes. *Biomaterials* 34 5813–5820. 10.1016/j.biomaterials.2013.04.026 23642535 PMC3660435

[B90] Zhang Y. S. Yue K. Aleman J. Mollazadeh-Moghaddam K. Bakht S. M. Yang J. (2017). 3D bioprinting for tissue and organ fabrication. *Ann. Biomed. Eng.* 45 148–163. 10.1007/s10439-016-1612-8 27126775 PMC5085899

[B91] Zimmermann W. H. Schneiderbanger K. Schubert P. Didié M. Münzel F. Heubach J. F. (2002). Tissue engineering of a differentiated cardiac muscle construct. *Circ. Res.* 90 223–230. 10.1161/hh0202.103644 11834716

[B92] Zislin V. Rosenfeld M. (2018). Impedance pumping and resonance in a multi-vessel system. *Bioengineering* 5:63. 10.3390/bioengineering5030063 30096933 PMC6164910

